# Optical Orientation of Excitons in a Longitudinal Magnetic Field in Indirect-Band-Gap (In,Al)As/AlAs Quantum Dots with Type-I Band Alignment

**DOI:** 10.3390/nano13040729

**Published:** 2023-02-14

**Authors:** T. S. Shamirzaev, A. V. Shumilin, D. S. Smirnov, D. Kudlacik, S. V. Nekrasov, Yu G. Kusrayev, D. R. Yakovlev, M. Bayer

**Affiliations:** 1 Rzhanov Institute of Semiconductor Physics, Siberian Branch of the Russian Academy of Sciences, 630090 Novosibirsk, Russia; 2 Novosibirsk State University, 630090 Novosibirsk, Russia; 3 Ioffe Institute, Russian Academy of Sciences, 194021 St. Petersburg, Russia; 4 Experimentelle Physik 2, Technische Universität Dortmund, 44227 Dortmund, Germany

**Keywords:** quantum dots, excitons, spins, optical orientation, hyperfine interaction, spin blockade

## Abstract

Exciton recombination and spin dynamics in (In,Al)As/AlAs quantum dots (QDs) with indirect band gap and type-I band alignment were studied. The negligible (less than 0.2 μeV) value of the anisotropic exchange interaction in these QDs prevents the mixing of the excitonic basis states and makes the formation of spin-polarized bright excitons possible under quasi-resonant, circularly polarized excitation. The recombination and spin dynamics of excitons are controlled by the hyperfine interaction between the electron and nuclear spins. A QD blockade by dark excitons was observed in the magnetic field, that eliminates the impact of nuclear spin fluctuations. A kinetic model which accounts for the population dynamics of the bright and dark exciton states as well as for the spin dynamics was developed to quantitatively describe the experimental data.

## 1. Introduction

Spin-dependent phenomena in semiconductor heterostructures are attractive from the viewpoints of both basic physics [1,2] and potential applications [3,4]. Semiconductor quantum dots (QDs) are of great interest as objects with long spin lifetimes of electrons and holes, as the key obstacle in spin-based quantum information processing is spin relaxation. Indeed, carrier localization slows down spin relaxation due to the suppression of the mechanisms determining the relaxation of freely moving charge carriers [5]. Therefore, the spin relaxation time of electrons localized in QDs can reach milliseconds, as confirmed experimentally [6].

A common approach to study spin dynamics is optical orientation provided by circularly polarized light [7]. Light delivers angular momentum to the electron spin system, inducing its polarization, which subsequently decays due to relaxation processes. Spin dynamics can be measured by monitoring the decay of the photoluminescence (PL) circular polarization degree [7]. However, this technique is not suitable to study the spin dynamics of excitons in direct-band-gap QDs at zero magnetic field. Axial symmetry breaking, which always occurs in experimentally available QDs, leads to the mixing of the bright pure spin exciton states via anisotropic exchange interaction [2,8]. Therefore, until recently, optical orientation at zero magnetic field was mainly used in QDs to study charged excitons (trions) formed, for example, from a pair of electrons and a hole [9]. In this case, the ground state of the trions is an electron spin singlet for which the exchange interaction with the hole vanishes [10,11].

We recently demonstrated the suppression of the anisotropic exchange interaction in indirect-band-gap (In,Al)As/AlAs QDs due to the small overlap of hole and electron wave functions in the momentum space, which prevents bright exciton mixing [12]. Additionally, the weak electron–nuclei interaction in the X valley makes the electrons in such QDs relatively robust against spin decoherence [13]. These features of exchange and hyperfine interactions led to the discovery of the dynamic electron spin polarization effect, which takes place under unpolarized optical excitation in magnetic fields of the order of a few milliteslas [14,15]. This effect was also later described for organic semiconductors [16] and moiré QDs [17], where it was recently observed experimentally [18].

In this paper, exciton recombination and spin dynamics in (In,Al)As/AlAs QDs with indirect band gap and type-I band alignment were studied in a longitudinal magnetic field under optical orientation. The magnetic fields were moderately weak, so all the Zeeman splittings of the spin states were much smaller than the thermal energy. These experimental conditions prevent the circular polarization of the exciton emission to be induced by the magnetic field [19,20,21,22,23]. Measuring the optical orientation in (In,Al)As/AlAs QDs with modulation of the sign of the circular polarization of the exciting light revealed a dependence of the PL circular polarization degree on the modulation frequency, which arose due to the long times of exciton recombination and spin relaxation in indirect-gap QDs. Different protocols for spin orientation measurement were compared, and the effect of QD blockade by dark excitons was found.

The paper is organized as follows: In Section 2, the studied heterostructures and used experimental techniques are described. In Section 3, we present the experimental data, including time-resolved unpolarized PL; PL under selective excitation at zero magnetic field; the recovery of PL circular polarization in longitudinal magnetic fields under continuous-wave (cw), circularly polarized excitation; and the effects of different excitation detection protocols. Then, in Section 4, the theory of exciton spin dynamics in QDs is presented and compared with the experiment.

## 2. Experimental Details

**Figure 1:**
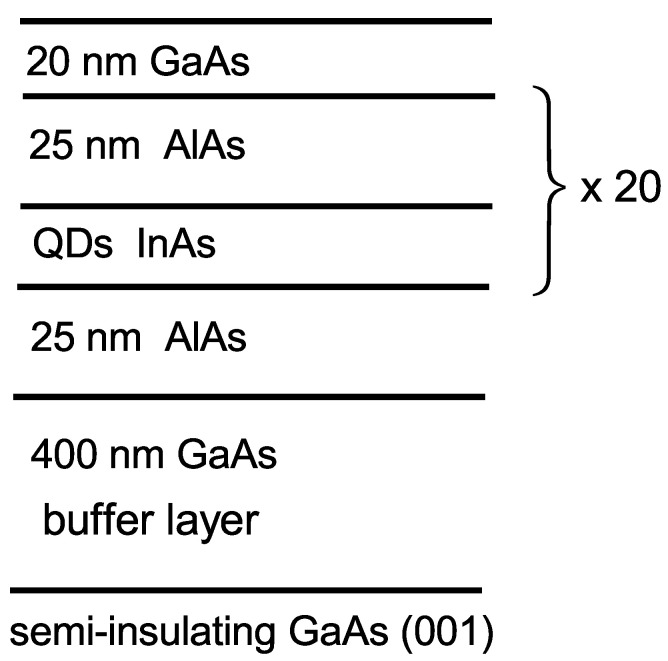
Schematic design of the studied heterostructure with QDs.

The studied self-assembled (In,Al)As QDs embedded in an AlAs matrix were grown using molecular-beam epitaxy on a semi-insulating, (001)-oriented GaAs substrate with a 400 nm thick GaAs buffer layer [24]. The structure contained 20 layers of undoped (In,Al)As/AlAs QDs sandwiched between 25 nm thick AlAs layers (see Figure 1). The nominal amount of deposited InAs was about 2.5 monolayers.

Lens-shaped QDs were formed at the temperature of 520 °C, with growth interruption time of 20 s. The distributions of the QD diameter and height were Gaussian, with averages of 15 nm and 4 nm, respectively. The full width at half maximum (FWHM) of the distribution of the diameters was 6.5 nm, which was 43% of the average diameter. The QD density was about $3\times 10^{10}$ cm${}^{-2}$ in each layer. A 20 nm thick GaAs cap layer protected the top AlAs barrier against oxidation. Further growth details are given in Ref. [24]. The interlayer distance and QD density were chosen to prevent electronic coupling between individual quantum dots [25,26]. The growth axis, *z*, coincided with the (001) crystallographic direction. Note that the band gap energy of the GaAs substrate was 1.52 eV and that of the AlAs barrier was 2.30 eV [27].

The sample was mounted strain free inside a cryostat with a variable temperature insert. The temperature was varied from *T* = 1.7 K up to 20 K. Magnetic fields in the millitesla range were generated by an electromagnet with accuracy better than 0.1 mT. The magnetic field direction coincided with the structure growth axis (*z*), with which the wave vector of the excitation light was also aligned (Faraday geometry).

PL was excited either non-resonantly, with the photon energy of a laser considerably exceeding the emission energies in the QD ensemble, or selectively, with laser energy tuned to a value within the inhomogeneously broadened exciton emission band of the QDs. Non-resonant excitation was provided by the third harmonic of a Q-switched Nd:YVO${}_{4}$ pulsed laser with photon energy of 3.49 eV, pulse duration of 5 ns, and repetition rate of 2 kHz. The excitation density was kept below 100 nJ/cm${}^{2}$ [28]. For selective excitation, a cw Ti:Sapphire laser with photon energy tunable in the spectral range from 1.50 to 1.75 eV was used.

For the time-resolved and time-integrated PL measurements, we used a gated charge-coupled-device camera synchronized with the laser via an external trigger signal. The time between the pump pulse and the start of PL recording, $t_{\mathrm{delay}}$, could be varied from 0 up to 1 ms. The duration of PL recording, i.e., gate window $t_{\mathrm{gate}}$, could be varied from 1 ns to 500 μs. The signal intensity and the time resolution of the setup depended on $t_{\mathrm{delay}}$ and $t_{\mathrm{gate}}$. The highest time resolution of the detection system was 1 ns.

For measuring the optical orientation (optical alignment) effects, the circular (linear) polarization of the excitation laser and that of PL emission were selected according to the corresponding combination of circular (linear) polarizers (Glan–Thompson prism) as well as quarter-wave (half-wave) plates. For the optical orientation measurement, the circular polarization degree of the PL induced by circularly polarized excitation, $\rho_{c}$, is defined as

(1) $$\begin{array}{c} \rho_{c}=\frac{I^{+/+}-I^{+/-}}{I^{+/+}+I^{+/-}}. \end{array}$$

where $I^{a/b}$ is the intensity of the $\sigma^{b}$-polarized PL component measured under $\sigma^{a}$-polarized excitation. Labels + and − correspond to right-hand and left-hand circular polarization, respectively. Note that in our experiments, the circular polarization degree induced by the external magnetic field was negligible, since the Zeeman splitting of the excitonic states was much smaller than the thermal energy.

In the optical alignment measurement, the linear polarization degree of the cw PL ($\rho_{l}$) induced by linearly polarized excitation was measured. The linear polarization degree is defined as

(2) $$\begin{array}{c} \rho_{l}=\frac{I^{0/0}-I^{0/90}}{I^{0/0}+I^{0/90}}, \end{array}$$

where $I^{a/b}$ are the PL intensities, with superscripts a/b corresponding to the direction of excitation/detection linear polarization. The “0” direction is parallel to the [110] crystallographic direction, and the “90” direction is parallel to the $\left[1\bar{1}0\right]$ direction.

**Figure 2:**
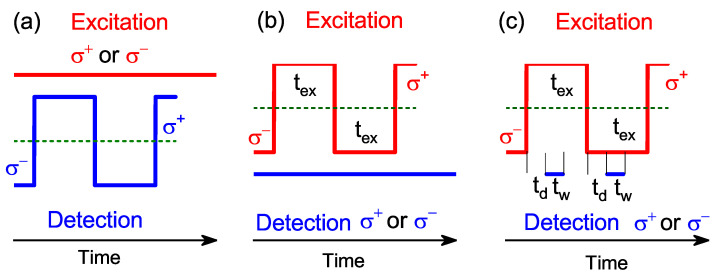
Protocols used for measurement of $\rho_{c}$: (**a**) cw circularly ($\sigma^{+}$ or $\sigma^{-}$) polarized excitation and modulation of polarization in the detection channel. (**b**) cw measurement of the emission in $\sigma^{+}$ or $\sigma^{-}$ polarization and modulation of polarization in the excitation channel with the period of 2$t_{ex}$. (**c**) Modulation of polarization in the excitation channel with the period of 2$t_{ex}$ and measurement in time window $t_{w}$ with delay $t_{d}$ after changing the excitation polarization.

Electron spin dynamics were investigated by measuring the PL polarization degree for optical orientation in longitudinal magnetic fields. In these experiments, PL was detected using a GaAs photomultiplier combined with a time-correlated photon-counting module. Three protocols were used: (i) cw circularly polarized excitation ($\sigma^{+}$ or $\sigma^{-}$ ) and measurement of $\rho_{c}$ using an acousto-optic quarter-wave modulator with modulation frequency $f_{m}=\frac{1}{2\times t_{\mathrm{ex}}}=50$ kHz, where $2\times t_{\mathrm{ex}}$ is the modulation period (Figure 2a); (ii) modulation of excitation polarization using an electro-optic half-wave modulator before the quarter-wave plate (with $f_{m}$ in the range from 1 up to 500 kHz) and cw measurement of the emission in $\sigma^{+}$ or $\sigma^{-}$ polarization (Figure 2b); (iii) the same excitation scenario as in protocol (ii) but measurement with a delay $t_{d}$ after changing the excitation polarization (from $\sigma^{+}$ to $\sigma^{-}$, and vice versa) in time window $t_{w}$ (Figure 2c).

## 3. Experimental Results

**Figure 3:**
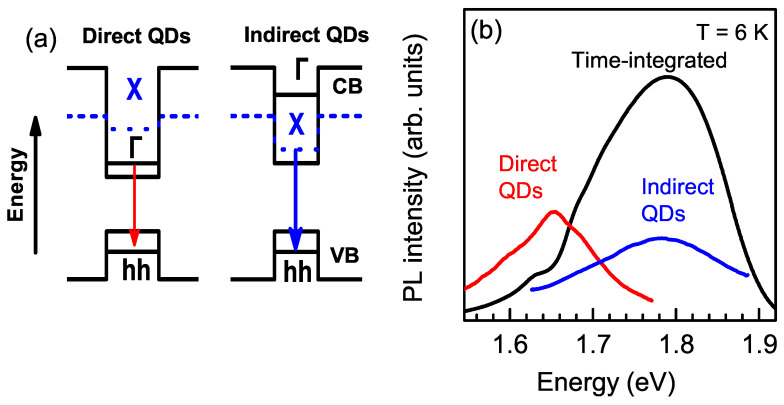
(**a**) Band diagrams of (In,Al)As/AlAs QDs with direct and indirect band structures. Arrows mark the optical transitions related to the decay of the ground-state exciton. (**b**) PL spectra of (In,Al)As/AlAs QDs measured under non-resonant excitation: time-integrated (black line); time-resolved for $t_{\mathrm{delay}}=1$ ns and $t_{\mathrm{gate}}=4$ ns (red) and for $t_{\mathrm{delay}}=1000$ ns and $t_{\mathrm{gate}}=1500$ ns (blue). T=6 K.

**Figure 4:**
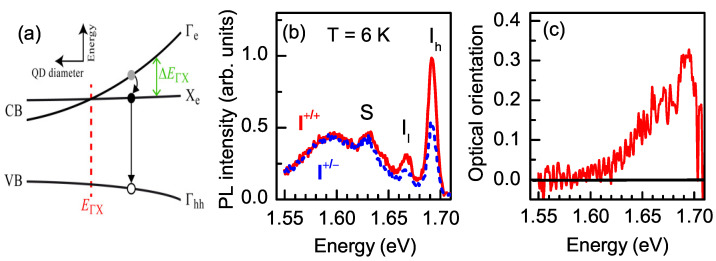
(**a**) Band alignment in QDs as function of dot diameter for the valence (VB) and conduction (CB) bands. The energy relaxation of a photoexcited electron from Γ to X and the subsequent recombination are indicated by the arrows. (**b**) PL spectra of (In,Al)As/AlAs QDs measured in $\sigma^{+}$ and $\sigma^{-}$ polarization under $\sigma^{+}$ circularly polarized excitation. $E_{\mathrm{exc}}=1.72$ eV, T=6 K. (**c**) Optical orientation calculated with the data shown in panel (**b**).

The dispersion of the QD size, shape, and composition within the ensemble led to the formation of (In,Al)As/AlAs QDs with different band structures [24], as shown in Figure 3a. The electron ground state changed from the Γ to the X valley with the decrease in dot diameter, while the heavy hole (hh) ground state remained at the Γ point (see Figure 4a). This corresponded to a change from a direct to an indirect band gap in momentum space, while type-I band alignment was preserved, that is, in both cases, electron and hole were spatially confined within the (In, Al)As QDs [24,28,29]. Change in the electron valley happens because the level in the Γ valley shifts to higher energies faster than that in the X valley with the decrease in QD size due to the smaller effective mass.

Recently, we demonstrated that the coexistence of (In,Al)As/AlAs QDs with direct and indirect band gaps within an ensemble results in a spectral dependence of the exciton recombination times. In momentum-direct QDs, excitons recombine within a few nanoseconds. On the contrary, momentum-indirect QDs are characterized by long decay times due to the small exciton oscillator strength [24,28,29,30,31,32,33,34]. Here, we used time-resolved PL to select the indirect-band-gap QDs.

### 3.1. Time-Resolved Unpolarized PL

The PL spectra of the (In,Al)As/AlAs QD ensemble measured under non-resonant excitation are shown in Figure 3b. The time-integrated spectrum (black line) has its maximum at 1.79 eV and extends from 1.5 to 1.9 eV, having FWHM of 190 meV. The large width of the emission band is due to the dispersion of the QD parameters, since the exciton energy depends on QD size, shape, and composition [24]. The PL band consists of the contributions from direct and indirect QDs, which becomes evident from the time-resolved PL spectra. When the spectrum was measured immediately after the laser pulse ($t_{\mathrm{delay}}=1$ ns and $t_{\mathrm{gate}}=4$ ns), the PL band was found to have the maximum at 1.65 eV and the FWHM of 120 meV (red line). For longer delays ($t_{\mathrm{delay}}=1000$ ns and $t_{\mathrm{gate}}=1500$ ns), the emission maximum shifts to 1.78 eV and broadens to 190 meV (blue line), rather similar to the time-integrated PL spectrum.

We recently demonstrated that after photoexcitation in the AlAs barriers, electrons and holes are captured into QDs within several picoseconds and that the capture probability does not depend on QD size and composition [35]. Therefore, all QDs in the ensemble (direct and indirect ones) became equally populated shortly after the excitation pulse. Thus, the exciton recombination dynamics were fast for direct QDs emitting mainly in the spectral range of 1.50–1.74 eV and slow for indirect QDs emitting in the range of 1.62–1.90 eV. The emissions of the direct and indirect QDs overlapped in the range of 1.62–1.74 eV.

### 3.2. PL under Selective Excitation at Zero Magnetic Field

In order to only excite a fraction of QDs with indirect band gap, we used selective excitation within the inhomogeneously broadened PL line. As a result, the PL band transformed into a spectrum with rather narrow lines [12]. PL spectra measured under $\sigma^{+}$ excitation at $E_{\mathrm{exc}}=1.72$ eV using co- and cross-polarized detection are shown in Figure 4b. As we showed in Ref. [12], the lines marked as $I_{l}$ and $I_{h}$ arose from exciton recombination in indirect QDs, while line *S* arose from a transition in QDs with Γ-X mixing of the electron states. Tuning the excitation energy allowed us to selectively excite different sub-ensembles of QDs.

**Figure 5:**
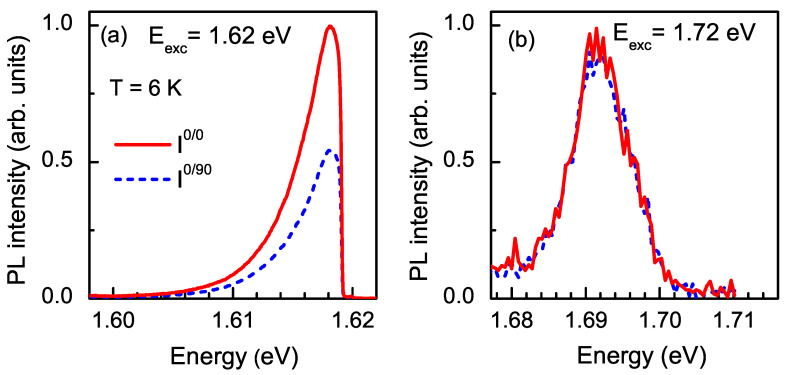
Linearly polarized PL spectra of (In,Al)As/AlAs QDs measured under linearly polarized excitation. T=6 K. (**a**) Direct-band-gap QDs. $E_{\mathrm{exc}}=1.62$ eV. (**b**) Indirect-band-gap QDs. $E_{\mathrm{exc}}=1.72$ eV.

The optical orientation across the PL spectrum under excitation at $E_{\mathrm{exc}}=1.72$ eV is shown in Figure 4c. The PL in the low-energy spectral region, which corresponds to exciton recombination in direct-band-gap QDs, demonstrated almost zero optical orientation. Contrary to that, indirect QDs (high-energy spectral region) demonstrated pronounced optical orientation that reached 0.3 (i.e., 30 %) at the maximum of the $I_{h}$ line (at 1.695 eV). Linearly polarized emission under linearly polarized excitation (optical alignment) was observed in direct QDs, but it was absent in indirect QDs, as shown in Figure 5a,b, respectively.

These results are explained by the exciton fine structure. The exciton is formed by a heavy hole with angular momentum projection $j_{z}=\pm 3/2$ and an electron with *s* = 1/2 spin. Accordingly, there are four exciton fine-structure states. The two bright exciton states are characterized by angular momentum projections $J_{z}=\pm 1$ onto growth axis *z*, and the two dark states have projections $J_{z}=\pm 2$. The breaking of the axial symmetry in direct QDs lifts the degeneracy of the bright exciton states and mixes them, so the following states emerge: $|X\rangle =\frac{1}{\sqrt{2}}\left(|+1\rangle +|-1\rangle \right)$ and $|Y\rangle =\frac{i}{\sqrt{2}}\left(|-1\rangle -|+1\rangle \right)$ [36]. A circularly polarized photon excites a superposition of states |X〉 and |Y〉, whose coherence is rapidly lost, destroying the optical orientation of the excitons [37]. Linearly polarized photons, by contrast, excite the pure states |X〉 and |Y〉 of the bright exciton, so that the linear polarization degree of the emission (optical alignment) is determined by the ratio of the exciton spin decoherence time to exciton lifetime $\tau_{R}$. The high value of optical alignment for direct QDs of more than 30% leads us to the conclusion that the spin decoherence time exceeds the recombination one, which is typical in direct-band-gap QDs [37]. For indirect-band-gap QDs, the anisotropic electron–hole exchange interaction is negligible due to the weak overlap of the wave functions of the X-electron and the Γ-hole in momentum space [38,39], so pure exciton spin states $J_{z}=\pm 1$ provide circularly polarized PL [12].

### 3.3. Optical Orientation in Longitudinal Magnetic Field

**Figure 6:**
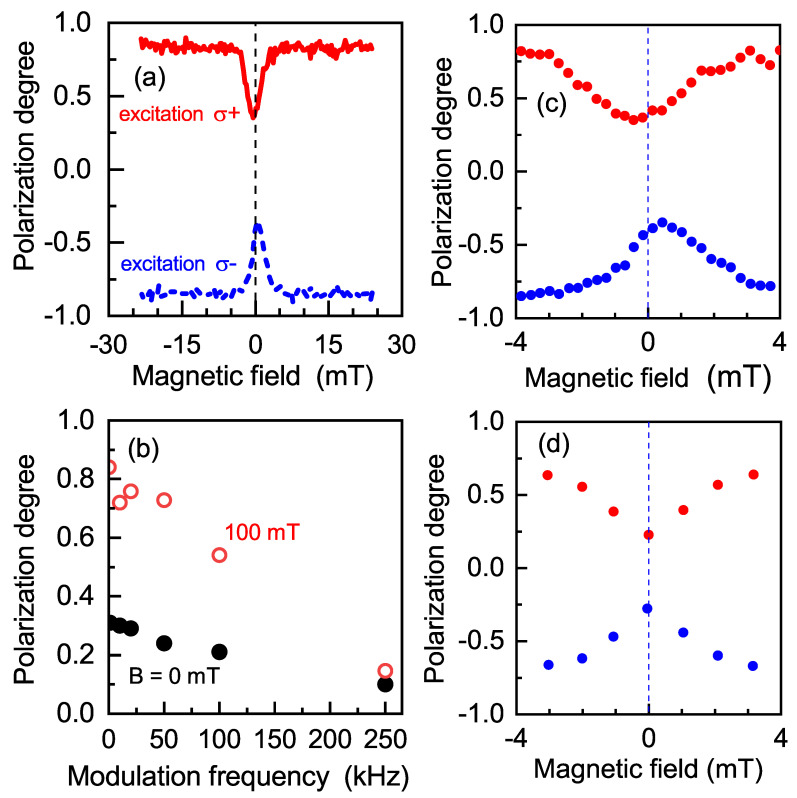
(**a**) Optical orientation in indirect-band-gap QDs at 1.695 eV for $E_{\mathrm{exc}}=1.72$ eV at T=6 K. Excitation with protocol (i): $\sigma^{+}$ (red solid line) and $\sigma^{-}$ (blue dashed line). (**b**) $\rho_{c}^{0}$ (black circles) and $\rho_{\mathrm{sat}}$ for *B* = 100 mT (red circles) as functions of the excitation polarization modulation frequency in protocol (ii). (**c**,**d**) Details of the PL circular polarization degree near zero external magnetic field for protocols (i) and (ii), respectively.

The optical orientation at the I${}_{h}$ line maximum (1.695 eV) under selective cw excitation at $E_{\mathrm{exc}}=1.72$ eV in protocol (i) (see Figure 2a) as function of the longitudinal magnetic field is shown in Figure 6a. One can see that at zero magnetic field, PL polarization degree $\rho_{c}^{0}=0.31$. Already in magnetic fields of a few milliteslas, $\rho_{c}\left(B\right)$ demonstrated a strong change. The optical orientation gradually increased with the increase in the magnetic field and saturated at $\rho_{\mathrm{sat}}$ = 0.84, which is about three times larger than $\rho_{c}^{0}$.

The shape of the polarization recovery curve (PRC) is described by a Lorentz curve, $\rho_{c}\left(B\right)=\rho_{c}^{0}+\left(\rho_{\mathrm{sat}}-\rho_{c}^{0}\right)/\left(1+\Delta_{\mathrm{PRC}}^{2}/B^{2}\right)$ [40], with a half width at half maximum of $\Delta_{\mathrm{PRC}}=1.8$ mT. We recently demonstrated that $\Delta_{\mathrm{PRC}}$ arises from the electron spin precession in the local fields created by nuclear spin fluctuations [41], which govern the electron spin dynamics in magnetic fields $B∼\Delta_{\mathrm{PRC}}$ [12,13,14].

We can estimate the anisotropic exchange interaction for indirect excitons as $\delta_{1}<\Delta_{\mathrm{PRC}}\mu_{B}g_{e}$ [12], where $\mu_{B}$ is the Bohr magneton and $g_{e}$ is the electron *g*-factor. With $g_{e}=2$ (which was measured using spin-flip Raman scattering [42] and optically detected magnetic resonance [43,44]), we obtain $\delta_{1}<0.2\;$μeV, which is indeed several orders of magnitude smaller than the $\delta_{1}$ of several hundreds of microelectronvolts observed in direct-band-gap (In,Al)As/AlAs QDs [8].

The increase in the PL polarization degree in a longitudinal magnetic field by a factor of about 3 (from $\rho_{c}^{0}=0.31$ to $\rho_{\mathrm{sat}}=0.84$) indicated that electron spin relaxation time $T_{1}$ was longer than the indirect exciton recombination time. Indeed, the electron spin in a QD undergoes Larmor precession around the effective frozen nuclear field, $B_{N}$, induced by nuclear spin fluctuations. The photogenerated spin-oriented electrons lose 2/3 of their spin polarization during time $T_{2}^{*}∼\hbar /\left(g_{e}\mu_{B}\Delta_{\mathrm{PRC}}\right)$, since $B_{N}$ has no preferential orientation and its direction varies from dot to dot in a QD ensemble. The remaining 1/3 of electron spin polarization is stabilized via the interaction with nuclear spins pointing along the orientation direction, i.e., the *z*-axis [12,13]. The deviation of $\rho_{\mathrm{sat}}$ from unity is the result of the loss of the electron and hole spin polarization during energy relaxation via the transition from the Γ-Γ exciton to the Γ-X exciton [13]. Note that the loss of electron spin polarization alone cannot describe $\rho_{\mathrm{sat}}<1$, because the hole spin uniquely defines the emitted photon polarization.

**Figure 7:**
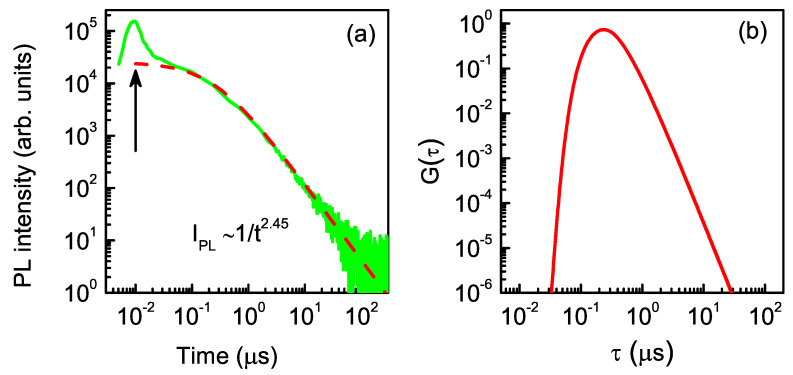
(**a**) PL dynamics measured at the detection energy of 1.695 eV. The excitation pulse at photon energy of 3.49 eV ends at t=10 ns (marked by the vertical arrow). The dashed curve is a fit obtained with the parameters given in the text. (**b**) Normalized exciton lifetime distribution function G(τ) corresponding to the QD sub-ensemble emitting at energy of 1.695 eV, obtained by fitting the PL dynamics.

Taking into account the exciton lifetime in the QDs, we can estimate the lower boundary for spin relaxation time $T_{1}$ of electrons in indirect-band-gap QDs in a longitudinal magnetic field, which eliminates the effect of the nuclear field on the electron spin dynamics. In order to determine the typical exciton lifetime in QDs, we measured the PL dynamics at the detection energy of 1.695 eV under non-resonant excitation, as shown in Figure 7a. The PL dynamics are plotted on a double-logarithmic scale, which is convenient to cover the wide range of scanned times and PL intensities.

The recombination dynamics demonstrate two distinctive stages: (i) a fast PL decay immediately after the excitation pulse corresponding to recombination in direct-band-gap QDs, since direct and indirect QDs coexist in this spectral region (see Figure 3); (ii) a further PL decay that can be described by the power-law function $I\left(t\right)∼{\left(1/t\right)}^{\alpha }$, as shown in our previous studies [24,28,43]. Such dynamics result from the superposition of multiple monoexponential decays with different times varying with the size, shape, and composition of indirect-band-gap QDs. It can be described by the following equation [28,45]:

(3) $$I\left(t\right)=\int_{0}^{\infty }G\left(\tau \right)\exp\left(-\frac{t}{\tau }\right)d\tau ,$$

where G(τ) is the distribution function of exciton recombination times τ. It has a rather simple form [28]:

(4) $$G\left(\tau \right)=\frac{C}{\tau^{\gamma }}\exp\left(-\frac{\tau_{0}}{\tau }\right).$$

where *C* is a constant and $\tau_{0}$ characterizes the maximum of the distribution of the exciton lifetimes. Parameter γ can be extracted directly from power-law decay (1/*t*)${}^{\gamma -1}$, presented in Figure 7a. Using the model approach suggested in our recent study [28], we obtained this distribution function by fitting the recombination dynamics in Figure 7a (see the dashed line). The fitting parameters were γ=3.45 and $\tau_{0}=0.25\;$μs. Distribution G(τ) is shown in Figure 7b. The typical recombination time, $\tau_{0}$, for excitons in the QD sub-ensemble emitting at 1.695 eV equaled 0.25 μs. Thus, the typical spin relaxation time, $T_{1}$, of electrons in indirect-band-gap QDs in a longitudinal magnetic field is longer than $\tau_{0}=0.25\;$μs.

### 3.4. Effect of Excitation Detection Protocol on Optical Orientation

The continuous excitation of localized electrons using circularly polarized light (as we applied in protocol (i)) can lead, via the Knight field, to the polarization of the nuclear spins, i.e., to dynamic nuclear polarization (DNP) [1,7,46]. The nuclear polarization degree is determined by the ratio between the spin transfer rate from electrons to the nuclei and the nuclear spin relaxation rate [1]. The nuclear spin relaxation times in A${}_{3}$B${}_{5}$ semiconductors can reach several seconds, and the Overhauser field of the polarized nuclei acting on the electrons can reach several teslas [7].

In our case, DNP manifested itself as a shift in the minimum of the PRC of 0.5 mT from the zero-field position (see Figure 6c). A change in excitation polarization (from $\sigma^{+}$ to $\sigma^{-}$) results in a change in the shift direction to the opposite one. Note that the value of the DNP-induced Overhauser field in our indirect-band-gap QDs was smaller than the typical one (about 10–20 mT) observed for direct-band-gap (In,Ga)As QDs under comparable excitation conditions [47]. The relatively weak DNP-induced Overhauser field in indirect-band-gap (In, Al)As QDs originated from two specific features of this system: (i) The long exciton lifetime reduced the rate of spin transfer from the electrons to the nuclei. Indeed, the number of electrons that have the possibility to transfer spin polarization to the nuclei in direct-band-gap systems, which have a typical exciton lifetime of a nanosecond, is about 10${}^{9}$ per second. On the other hand, in systems with indirect band gap, where the exciton lifetime is about a microsecond, this number decreases by several orders of magnitude. (ii) As recently shown, the hyperfine interaction constant for an electron in the X valley of (In, Al)As QDs with As nuclei is about two times and, with In and Al nuclei, about two orders of magnitude smaller than that for an electron in the Γ valley [13]. Thus, the Overhauser field induced by polarized nuclei in indirect QDs is several times smaller than the one in direct-band-gap QDs even for a similar nuclear spin polarization degree.

A common technique for DNP suppression during optical orientation is the modulation of the helicity of the exciting light [7]. We used this technique (excitation corresponding to protocol (ii)) for the measurement of the PRCs. Figure 6d demonstrates the absence of a shift in the PRCs at $f_{m}=10$ kHz, which evidences DNP suppression.

However, a strong difference in optical orientation occurred when using excitation protocol (ii) compared with protocol (i). $\rho_{c}^{0}$ and $\rho_{\mathrm{sat}}$ for B=100 mT are shown in Figure 6b as functions of the modulation frequency of excitation polarization. One can see that $\rho_{c}^{0}$ decreased with the increase in $f_{m}$ from 0.31 at $f_{m}=0$ to 0.10 at $f_{m}=250$ kHz. $\rho_{\mathrm{sat}}$, equal to 0.84 at $f_{m}=0$, decreased down to 0.15 at $f_{m}=250$ kHz.

**Figure 8:**
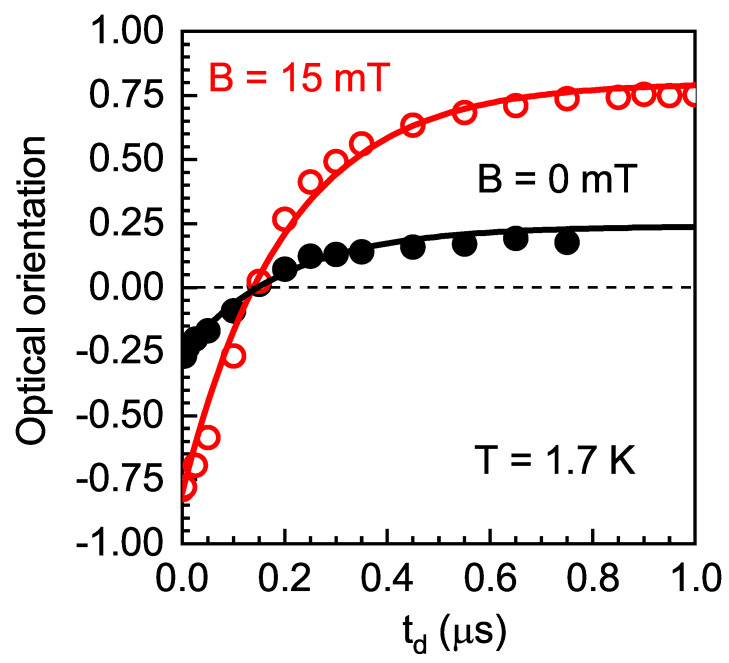
Optical orientation $\rho_{c}^{0}$ (filled black circles) and $\rho_{\mathrm{sat}}$ for B=15 mT (open red circles) as functions of delay time $t_{d}$, measured at $t_{\mathrm{ex}}=1\;$μs and $t_{w}=0.05\;$μs. T=1.7 K. $t_{d}=0$ corresponds to a change in excitation polarization from $\sigma^{-}$ to $\sigma^{+}$. The solid lines show fits after Equation (5) with the parameters given in the text.

In order to understand these results, we used protocol (iii) (see Figure 2c) with different excitation times $t_{\mathrm{ex}}$, delay times $t_{d}$, and measurement time windows $t_{w}$. The dependence of $\rho_{c}^{0}$($t_{d}$) and $\rho_{\mathrm{sat}}$($t_{d}$) for B=15 mT, measured at $t_{\mathrm{ex}}=1\;$μs and $t_{w}=0.05\;$μs, is shown in Figure 8. One can see that both $\rho_{c}^{0}$ and $\rho_{\mathrm{sat}}$, at zero delay time, corresponding to the change in excitation polarization from $\sigma^{-}$ to $\sigma^{+}$, surprisingly, were negative (i.e., they were dominated by the counter-polarized $I^{+/-}$ PL component) and equal to −0.75 (−0.25) for $\rho_{\mathrm{sat}}$ ($\rho_{c}^{0}$). When the delay time increased, the optical orientation decreased to zero at $t_{d}=0.15\;$μs. A further increase in $t_{d}$ changed the polarization to positive values (dominated by the co-polarized $I^{+/+}$ PL component), and the polarization degree increased to +0.75 (+0.25) for $\rho_{\mathrm{sat}}$ ($\rho_{c}^{0}$) at $t_{d}=1\;$μs.

The change in excitation polarization upon modulation of the exciting light in protocols (ii) and (iii) occurred on timescales much shorter than the indirect exciton lifetime. This resulted in a situation where after the change in excitation polarization, a fraction of QDs was still occupied by excitons created in the previous half period of excitation with the corresponding direction of spin polarization, while the other fraction of QDs began to become occupied with excitons of opposite spin polarization. Both types of excitons recombined, simultaneously emitting oppositely polarized photons. The ratio of oppositely polarized exciton concentrations changed over time. Using continuous detection, we measured the integral from all of these processes, which varied with the modulation frequency of excitation polarization (see Figure 6b).

We can describe the dependence of the optical orientation on the delay after changing the excitation polarization as follows:

(5) $$\rho_{c}\left(t\right)=\frac{\left[I^{+/+}\left(t\right)+I^{-/+}\left(t\right)\right]-\left[I^{+/-}\left(t\right)+I^{-/-}\left(t\right)\right]}{\left[I^{+/+}\left(t\right)+I^{-/+}\left(t\right)\right]+\left[I^{+/-}\left(t\right)+I^{-/-}\left(t\right)\right]}=\rho_{c}^{e}\left[1-2\exp\left(-t/\tau \right)\right],$$

where $\rho_{c}^{e}$ is the circular polarization degree at the end of the excitation period for $t=t_{\mathrm{ex}}$. One can see that the experimental data in Figure 8 can be well fitted using Equation (5) with exciton recombination time τ=0.21μs, which is in reasonable agreement with $\tau_{0}=0.25\;$μs, obtained with the PL dynamics measurements in Section 3.3.

**Figure 9:**
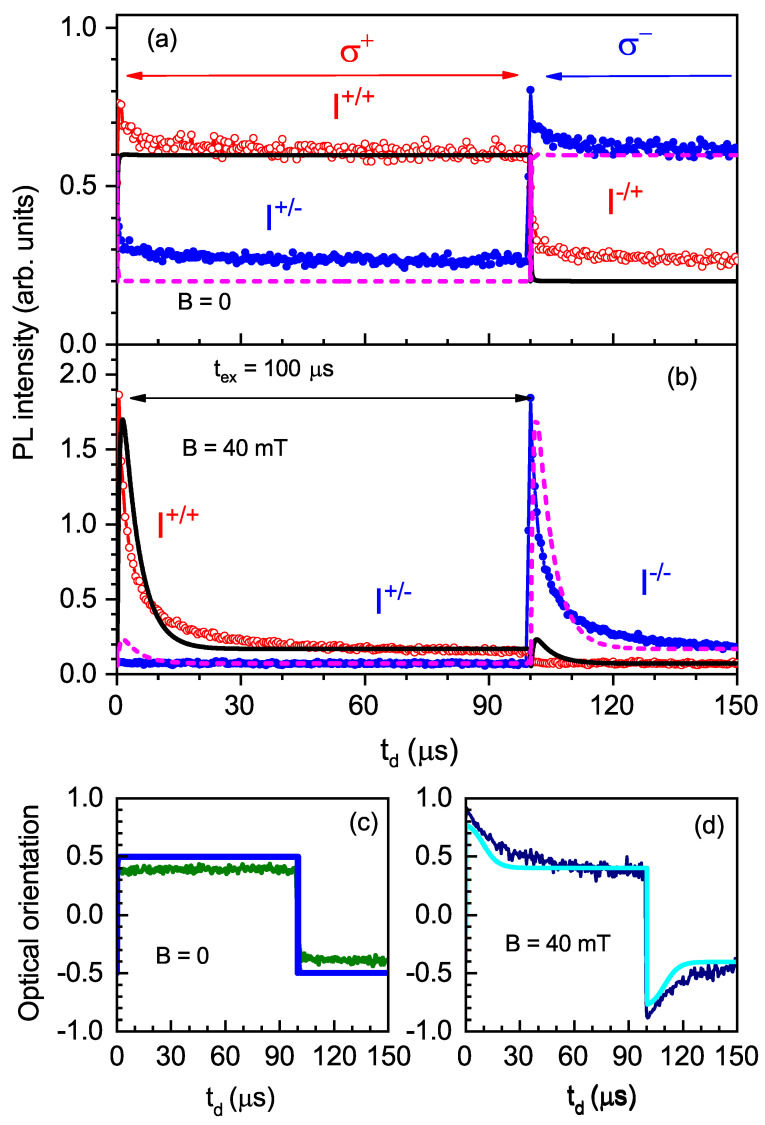
Intensities of PL components labeled in the figure as functions of delay time $t_{d}$ for (**a**) B=0 mT and (**b**) B=40 mT, measured at $t_{\mathrm{ex}}=100\;$μs and $t_{w}=2\;$μs. T=1.7 K. The optical orientation corresponding to these functions is presented in panel (**c**) for B=0 mT and panel (**d**) for B=40 mT. The theoretical simulations with the parameters given in the text are shown with the solid and dashed lines for the corresponding external magnetic fields.

In order to study the spin dynamics on time scales that strongly exceed the exciton lifetimes, we measured the intensities of the co- ($I^{+/+}$) and counter-polarized ($I^{+/-}$) PL components for $t_{\mathrm{ex}}=100\;$μs and $t_{w}=2\;$μs. The results of these measurements at zero magnetic field and in a magnetic field of 40 mT (which corresponds to $\rho_{\mathrm{sat}}$) are shown in Figure 9 as functions of $t_{d}$. At zero magnetic field, both co- and counter-polarized PL component intensities, after the short transient process following the change in the excitation polarization, had identical temporal dependence (see Figure 9a), resulting in constant polarization (see Figure 9c). However, this dependence drastically changed in the magnetic field. The intensity of the counter-polarized PL component did not depend on delay time, while the co-polarized PL component strongly increased with the change in excitation polarization, namely, by an order of magnitude (Figure 9b), and then decayed with the increase in $t_{d}$. Thus, the PL polarization degree followed the intensity of the co-polarized component (see Figure 9d).

**Figure 10:**
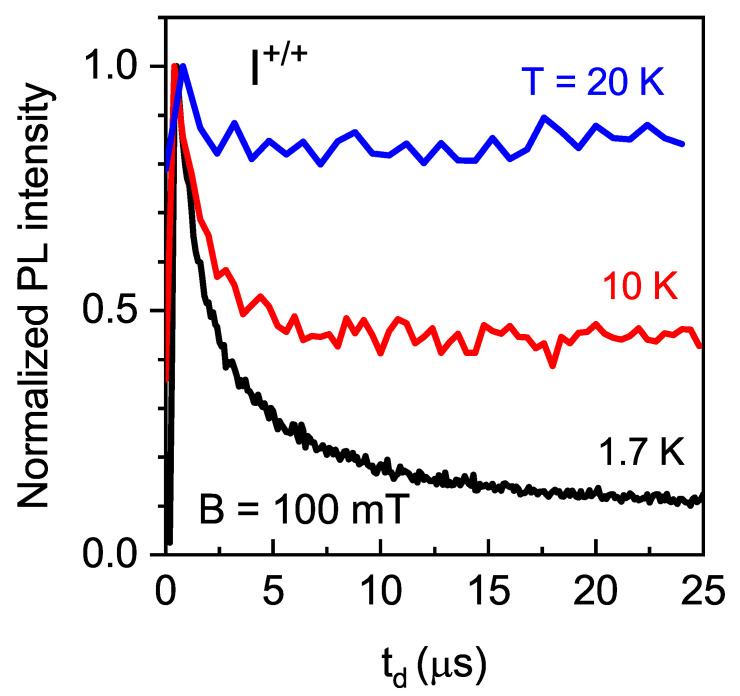
Intensity of co-polarized ($I^{+/+}$) PL component as function of delay time $t_{d}$ for B=100 mT, measured with $t_{\mathrm{ex}}=25\;$μs, $t_{w}=2\;$μs, and different temperatures.

Finally, Figure 10 shows that at higher temperatures, the decay of the co-polarized PL ($I^{+/+}$) with delay time $t_{d}$ became weaker.

In conclusion of this section, we summarize the most important experimental findings. The magnetic field dependence of the optical orientation in (In,Al)As/AlAs QDs strongly changed with the measurement protocol:

(i) The optical orientation depends on the modulation frequency of the excitation polarization.
(ii) Measurement of the $\sigma^{+}$- and $\sigma^{-}$-polarized PL components in a short time window $t_{w}$ with delay $t_{d}$ after changing the excitation polarization (from $\sigma^{+}$ to $\sigma^{-}$, and vice versa) allows us to reveal the lifetime and other features of the exciton spin dynamics in indirect QDs.
(iii) At zero magnetic field, both the co- and counter-polarized PL component intensities have identical temporal dependence, while in a magnetic field with a strength exceeding the fluctuations of the nuclear field, the intensity of the co-polarized PL component strongly increases after the change in the excitation polarization and then decays with the increase in $t_{d}$. However, the intensity of the counter-polarized PL component does not depend on the delay time.
(iv) The decrease in co-polarized PL component intensity with delay time $t_{d}$ in a magnetic field disappears with the increase in temperature.

## 4. Discussion

The most surprising experimental result is shown in Figure 9. Figure 9b evidences that after switching the excitation polarization, the intensity of the co-polarized emission, $I^{+/+}$ or $I^{-/-}$, strongly changed at $t_{d}∼10\;$μs. This happened in a magnetic field of 40 mT, while at zero magnetic field, there were no such changes, as demonstrated in Figure 9a.

In some systems, the decrease in PL intensity may be related to the suppression of the mixing of dark and bright excitons [17,48] after DNP. However, in our case, we show in Section 3.3 that the splitting between the bright and dark excitons was small, so at least half of the excitons created by means of quasi-resonant excitation were bright. In this case, the suppression of the mixing between bright and dark excitons cannot explain the decrease in PL intensity by an order of magnitude.

**Figure 11:**
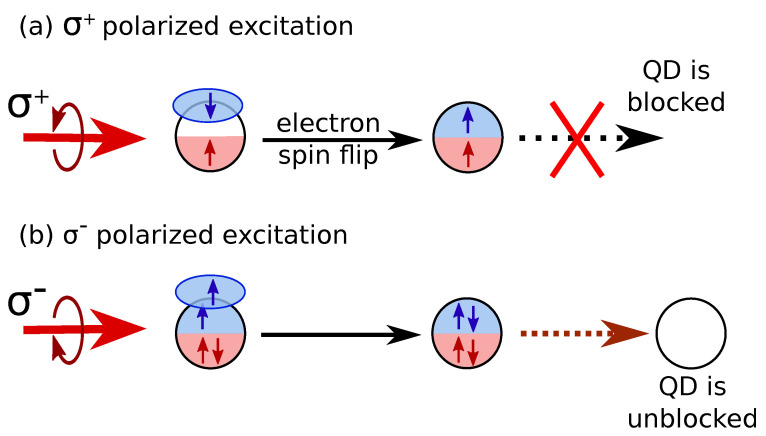
(**a**) Dark exciton in QD blocks creation of another exciton under $\sigma^{+}$-polarized excitation and lives for a long time. (**b**) Under $\sigma^{-}$-polarized excitation, another exciton can be created; then, two excitons recombine, leaving an empty QD.

We suggest that the observed effect is related to the blockade of QDs by dark excitons. Let us qualitatively describe the mechanism of decrease in co-polarized PL intensity over time. In a strong-enough longitudinal magnetic field, the nuclei-induced mixing between bright and dark excitons is negligible. In this case, $\sigma^{+}$ excitation mostly creates bright excitons; however, due to electron spin relaxation, dark excitons can also be created. These excitons have long lifetimes that are controlled by hole spin-flip rate $\gamma_{h}$. While each QD can can be occupied by a single exciton with a given spin only, dark excitons can accumulate in the ensemble and occupy a significant fraction of QDs, leading to the suppression of PL intensity (Figure 11a).

When excitation polarization is switched from $\sigma^{+}$ to $\sigma^{-}$, the possibility appears for QDs to capture a second photon and form a biexciton. Fast biexciton recombination returns the blocked QDs to the optically active state, and PL intensity recovers (Figure 11b). The Pauli exclusion principle forbids biexciton formation with the initial excitation polarization.

This phenomenon occurs when the applied magnetic fields are strong enough. At zero magnetic field, bright and dark excitons are effectively mixed by the random nuclear field, so all of the four exciton types can quickly recombine radiatively. As a result, PL intensity does not strongly change over time, as shown in Figure 9a.

With the increase in temperature, the electron spin relaxation accelerates, so the effect of the PL intensity decrease over time disappears, in agreement with Figure 10.

### 4.1. Theory of QD Blockade

In this section, we give a detailed model of the QD blockade by dark excitons.

**Figure 12:**
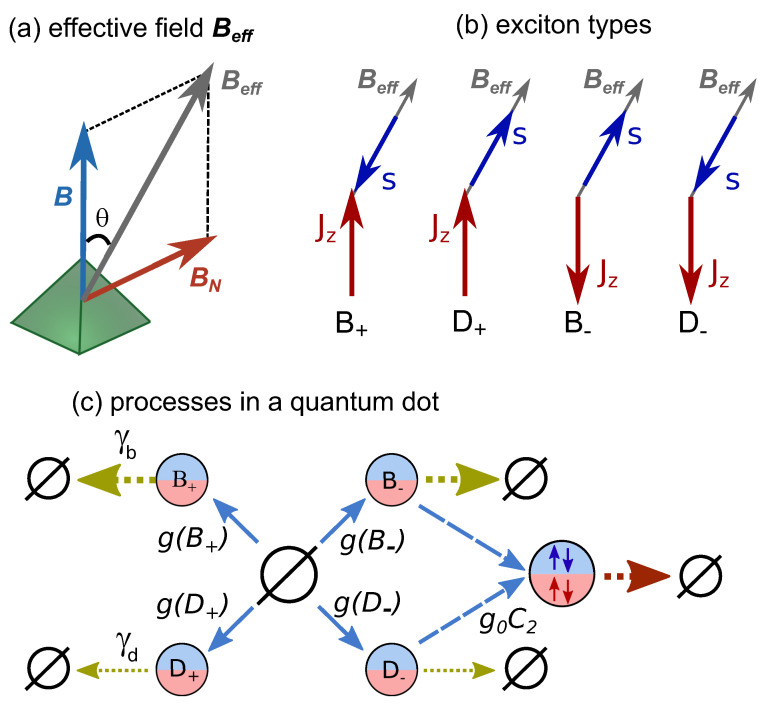
(**a**) Total effective magnetic field $B_{\mathrm{eff}}$ acting on an electron composed of random nuclear field $B_{N}$ and external field B parallel to growth axis *z*. (**b**) The four exciton eigenstates with the electron spin (blue arrow) along or opposite to the effective magnetic field (gray arrow) and the hole spin (red arrow) along or opposite to the *z*-axis. (**c**) Possible transitions between QD states during $\sigma^{+}$ excitation (see text for details), where ⌀ denotes the empty QD state.

Under quasi-resonant excitation by $\sigma^{+}$ ($\sigma^{-}$)-polarized light, bright excitons are created in QDs with a spin-up (-down) heavy hole and a spin-down (-up) electron. We assume that shortly after excitation, electron and hole can flip their spins with probabilities $f_{e}$ and $f_{h}$, respectively, during exciton relaxation. As a result, the occupancies of the electron spin-up and spin-down states are $f_{e}$ ($1-f_{e}$) and $1-f_{e}$ ($f_{e}$), which is similarly true for the hole spin states. The spin flips may be related to the electron–hole exchange interaction in the momentum-direct exciton state or to the electron–phonon interaction during electron energy relaxation. For the ground state, we describe the electron spin dynamics using the precession in the effective magnetic field, $B_{\mathrm{eff}}$, composed of external field B and random nuclear field $B_{N}$ [14,15] (see Figure 12a). We assume the nuclear field to be quasi-static and Gaussian-distributed as $\propto \exp(-B_{N}^{2}/\Delta_{B}^{2})$ [2,41,49], with parameter $\Delta_{B}$ determining the dispersion, neglecting the anisotropy of the hyperfine interaction [13] and the intervalley hyperfine interaction [50]. Due to light polarization modulation, DNP is absent [51], so we neglect this, as well as the nuclear spin dynamics, which in principle can take place on a sub-millisecond time scale [52].

We assume the electron spin precession (the typical period is of the order of 10 ns) to be faster than the exciton recombination, so the direction of $B_{\mathrm{eff}}$ defines the appropriate quantization axis for the electron spin. We denote the exciton states with the electron spin along or opposite to the direction of $B_{\mathrm{eff}}$ and the hole spin-up or -down as $B_{\pm }$ and $D_{\pm }$, respectively (see Figure 12b). In a strong longitudinal magnetic field, $B_{z}\gg B_{N}$, the effective magnetic field is almost parallel to the *z*-axis, so $B_{\pm }$ and $D_{\pm }$ are quasi-bright and quasi-dark states, respectively.

As described above, we consider the generation of all four exciton states starting from an empty QD. Under $\sigma^{+}$ excitation, the corresponding generation rates have the forms

(6) $$g\left(B_{+}\right)=g_{0}\left[\left(1-f_{h}\right)\left(1-f_{e}\right)\frac{1+\cos\theta }{2}+\left(1-f_{h}\right)f_{e}\frac{1-\cos\theta }{2}\right],$$

(7) $$g\left(D_{+}\right)=g_{0}\left[\left(1-f_{h}\right)f_{e}\frac{1+\cos\theta }{2}+\left(1-f_{h}\right)\left(1-f_{e}\right)\frac{1-\cos\theta }{2}\right],$$

(8) $$g\left(B_{-}\right)=g_{0}\left[f_{h}f_{e}\frac{1+\cos\theta }{2}\right.\left.+f_{h}\left(1-f_{e}\right)\frac{1-\cos\theta }{2}\right],$$

(9) $$g\left(D_{-}\right)=g_{0}\left[f_{h}\left(1-f_{e}\right)\frac{1+\cos\theta }{2}+f_{h}f_{e}\frac{1-\cos\theta }{2}\right],$$

where $g_{0}$ is the pumping rate and θ is the angle between $B_{\mathrm{eff}}$ and the *z*-axis (see Figure 12a). Under $\sigma^{-}$ excitation, the subscripts of $D_{\pm }$ and $B_{\pm }$ should be flipped.

We take into account the radiative recombination of bright excitons with rate $\gamma_{r}=1/\tau$. In analogy with the generation rates, we find the rates of $B_{\pm }$ and $D_{\pm }$ exciton recombination as

(10) $$\gamma_{b}=\gamma_{r}\frac{1+\cos\theta }{2},\;\gamma_{d}=\gamma_{r}\frac{1-\cos\theta }{2},$$

respectively (see Figure 12c). We also consider the possibility of a hole spin flip with rate $\gamma_{h}$.

In addition to that, we allow biexciton formation to occur in a QD, as shown in Figure 12c. We assume that under $\sigma^{\pm }$ excitation, it can be formed from the $B_{\mp }$ and $D_{\mp }$ excitons only with rate $g_{0}C_{2}$ due to the Pauli exclusion principle for the heavy hole spin. The biexciton recombination rate is assumed to be higher than all other recombination rates for simplicity; this, however, hardly affects the results. The biexciton resonance in (In,Al)As/AlAs QDs is detuned from the exciton one [24]; therefore, biexciton PL is not detected. However, after biexciton recombination, the QD can be excited once again, so it becomes optically active. Thus, the role of biexciton generation is to facilitate dark exciton recombination and to unblock the QDs after changes in excitation polarization (see Figure 11).

The kinetic equations for this model are

(11) $$\frac{dn(B_{\pm })}{dt}=g\left(B_{\pm }\right)n\left(⌀\right)+\frac{\gamma_{h}}{2}\left[n\left(D_{\mp }\right)-n\left(B_{\pm }\right)\right]-\gamma_{b}n\left(B_{\pm }\right)-g_{0}C_{2}\frac{1\mp \sigma }{2}n\left(B_{\pm }\right),$$

(12) $$\frac{dn(D_{\pm })}{dt}=g\left(D_{\pm }\right)n\left(⌀\right)+\frac{\gamma_{h}}{2}\left[n\left(B_{\mp }\right)-n\left(D_{\pm }\right)\right]-\gamma_{d}n\left(D_{\pm }\right)-g_{0}C_{2}\frac{1\mp \sigma }{2}n\left(D_{\pm }\right),$$

where $n(B_{\pm })$ and $n(D_{\pm })$ are the occupancies of the corresponding excitonic states and n(⌀) is the probability for a QD to be unoccupied. Due to the assumption of a fast biexciton recombination rate, $n\left(⌀\right)=1-n\left(B_{+}\right)-n\left(B_{-}\right)-n\left(D_{+}\right)-n\left(D_{-}\right)$. σ=± denotes the $\sigma^{\pm }$ polarization of light. The processes described by these kinetic equations are shown in Figure 12c for σ=+1.

The intensities of $\sigma^{\pm }$ PL are given by

(13) $$I^{\pm }\propto \gamma_{r}\langle \frac{1+\cos\theta }{2}n\left(B_{\pm }\right)+\frac{1-\cos\theta }{2}n\left(D_{\pm }\right)\rangle ,$$

where the angular brackets denote the averaging over the nuclear field distribution.

### 4.2. Modeling of Experimental Results

To describe the experimental data with this model, we numerically calculated the PL intensity and polarization as functions of delay time $t_{d}$ for B=0 mT and B=40 mT. The averaging was performed over 100 random realizations of $B_{N}$. The comparison between the theoretical and experimental results is shown in Figure 9. The best fit was obtained using the parameters of $g_{0}=2.2\;$μs^−1^, $\gamma_{r}=13\;$μs^−1^, $\gamma_{h}=0.035\;$μs^−1^, $C_{2}=0.5$, and $\Delta_{B}=0.33\;\mathrm{mT}$. Note that the latter parameter was not determined reliably from the fit, and it should be smaller than 40 mT. The spin-flip probabilities were $f_{h}=0.3$ and $f_{e}=0.25$. One can see that the agreement between theory and experiment is good.

The most reliably determined parameter was $\gamma_{h}$, because it describes the decrease in the intensity over time in a strong magnetic field. We also note that the obtained value of $\gamma_{r}$ agrees in the order of magnitude with the radiative lifetimes independently determined in Section 3.3 and Section 3.4. A specific feature of our model is that even in strong magnetic fields, the hyperfine interaction plays a role, because it can produce the recombination rate of the quasi-dark excitons comparable to the slow hole spin relaxation rate. In principle, there can also be non-radiative recombination and electron spin flips, which have the same effect. Another feature of the model is the absence of the electron–hole exchange interaction, which supports the previous suggestion that it is weak [13,14].

## 5. Conclusions

Exciton recombination and spin dynamics in indirect-band-gap (In,Al)As/AlAs QDs with type-I band alignment were studied in a longitudinal magnetic field by means of optical orientation. We have demonstrated that the commonly used technique of measuring the optical orientation based on the modulation of the excitation polarization with continuous-wave detection gives ambiguous results, which depend on the modulation frequency due to long exciton recombination and spin relaxation times in this system. A technique based on measuring with a delay after the change in excitation polarization is proposed for overcoming this problem. A QD blockade by dark excitons was revealed using this technique. The experimental findings could be quantitatively described with a theoretical model accounting for the population dynamics of the bright and dark exciton states as well as biexciton formation in QDs.

## Data Availability

The data presented in this study are available on request from the corresponding author.

## References

[1] Dyakonov M.I. (2008). Spin Physics in Semiconductors.

[2] Glazov M.M. (2018). Electron and Nuclear Spin Dynamics in Semiconductor Nanostructures.

[3] Fert A. (2008). Nobel Lecture: Origin, development, and future of spintronics. Rev. Mod. Phys..

[4] Bader S.D., Parkin S.S.P. (2010). Spintronics. Annu. Rev. Condens. Matter Phys..

[5] Khaetskii A.V., Nazarov Y.V. (2000). Spin relaxation in semiconductor quantum dots. Phys. Rev. B.

[6] Kroutvar M., Ducommun Y., Heiss D., Bichler M., Schuh D., Abstreiter G., Finley J.J. (2004). Optically programmable electron spin memory using semiconductor quantum dots. Nature.

[7] Meier F., Zakharchenja B.P. (1984). Optical Orientation.

[8] Rautert J., Rakhlin M.V., Belyaev K.G., Shamirzaev T.S., Bakarov A.K., Toropov A.A., Mukhin I.S., Yakovlev D.R., Bayer M. (2019). Anisotropic exchange splitting of excitons affected by Γ-X mixing in (In,Al)As/AlAs quantum dots: Microphotoluminescence and macrophotoluminescence measurements. Phys. Rev. B.

[9] Taylor M.W., Spencer P., Murray R. (2015). Negative circular polarization as a universal property of quantum dots. Appl. Phys. Lett..

[10] Dunker D., Shamirzaev T.S., Debus J., Yakovlev D.R., Zhuravlev K.S., Bayer M. (2012). Spin relaxation of negatively charged excitons in (In,Al)As/AlAs quantum dots with indirect band gap and type-I band alignment. Appl. Phys. Lett..

[11] Shamirzaev T.S., Yakovlev D.R., Kopteva N.E., Kudlacik D., Glazov M.M., Krechetov A.G., Gutakovskii A.K., Bayer M. (2022). Spin dynamics of charged excitons in ultrathin (In,Al)(Sb,As)/AlAs and Al(Sb,As)/AlAs quantum wells with an indirect band gap. Phys. Rev. B.

[12] Rautert J., Shamirzaev T.S., Nekrasov S.V., Yakovlev D.R., Klenovský P., Kusrayev Y.G., Bayer M. (2019). Optical orientation and alignment of excitons in direct and indirect band gap (In,Al)As/AlAs quantum dots with type-I alignment. Phys. Rev. B.

[13] Kuznetsova M.S., Rautert J., Kavokin K.V., Smirnov D.S., Yakovlev D.R., Bakarov A.K., Gutakovskii A.K., Shamirzaev T.S., Bayer M. (2020). Electron-nuclei interaction in the X valley of (In,Al)As/AlAs quantum dots. Phys. Rev. B.

[14] Smirnov D.S., Shamirzaev T.S., Yakovlev D.R., Bayer M. (2020). Dynamic Polarization of Electron Spins Interacting with Nuclei in Semiconductor Nanostructures. Phys. Rev. Lett..

[15] Shamirzaev T.S., Shumilin A.V., Smirnov D.S., Rautert J., Yakovlev D.R., Bayer M. (2021). Dynamic polarization of electron spins in indirect band gap (In,Al)As/AlAs quantum dots in a weak magnetic field: Experiment and theory. Phys. Rev. B.

[16] Shumilin A.V. (2022). Dynamic spin polarization in organic semiconductors with intermolecular exchange interaction. Phys. Rev. B.

[17] Smirnov D.S. (2021). Dynamic valley polarization in moiré quantum dots. Phys. Rev. B.

[18] Wang X., Xiao C., Park H., Zhu J., Wang C., Taniguchi T., Watanabe K., Yan J., Xiao D., Gamelin D.R. (2022). Light-induced ferromagnetism in moiré superlattices. Nature.

[19] Ivchenko E.L. (2018). Magnetic circular polarization of exciton photoluminescence. Phys. Solid State.

[20] Shamirzaev T.S., Debus J., Yakovlev D.R., Glazov M.M., Ivchenko E.L., Bayer M. (2016). Dynamics of exciton recombination in strong magnetic fields in ultrathin GaAs/AlAs quantum wells with indirect band gap and type-II band alignment. Phys. Rev. B.

[21] Shamirzaev T.S., Rautert J., Yakovlev D.R., Debus J., Gornov A.Y., Glazov M.M., Ivchenko E.L., Bayer M. (2017). Spin dynamics and magnetic field induced polarization of excitons in ultrathin GaAs/AlAs quantum wells with indirect band gap and type-II band alignment. Phys. Rev. B.

[22] Shamirzaev T.S., Rautert J., Yakovlev D.R., Bayer M. (2021). Exciton recombination and spin relaxation in strong magnetic fields in ultrathin (In,Al)As/AlAs quantum wells with indirect band gap and type-I band alignment. Phys. Rev. B.

[23] Shamirzaev T.S., Yakovlev D.R., Bakarov A.K., Kopteva N.E., Kudlacik D., Gutakovskii A.K., Bayer M. (2020). Recombination and spin dynamics of excitons in thin (Ga,Al)(Sb,As)/AlAs quantum wells with an indirect band gap and type-I band alignment. Phys. Rev. B.

[24] Shamirzaev T.S., Nenashev A.V., Gutakovskii A.K., Kalagin A.K., Zhuravlev K.S., Larsson M., Holtz P.O. (2008). Atomic and energy structure of InAs/AlAs quantum dots. Phys. Rev. B.

[25] Shamirzaev T.S., Gilinsky A.M., Kalagin A.K., Toropov A.I., Gutakovskii A.K., Zhuravlev K.S. (2006). Strong sensitivity of photoluminescence of InAs/AlAs quantum dots to defects: Evidence for lateral inter-dot transport. Semicond. Sci. Technol..

[26] Shamirzaev T.S., Abramkin D.S., Dmitriev D.V., Gutakovskii A.K. (2010). Nonradiative energy transfer between vertically coupled indirect and direct bandgap InAs quantum dots. Appl. Phys. Lett..

[27] Vurgaftman I., Meyer J.R., Ram-Mohan L.R. (2001). Band parameters for III–V compound semiconductors and their alloys. J. Appl. Phys..

[28] Shamirzaev T.S., Debus J., Abramkin D.S., Dunker D., Yakovlev D.R., Dmitriev D.V., Gutakovskii A.K., Braginsky L.S., Zhuravlev K.S., Bayer M. (2011). Exciton recombination dynamics in an ensemble of (In,Al)As/AlAs quantum dots with indirect band-gap and type-I band alignment. Phys. Rev. B.

[29] Shamirzaev T.S., Nenashev A.V., Zhuravlev K.S. (2008). Coexistence of direct and indirect band structures in arrays of InAs/AlAs quantum dots. Appl. Phys. Lett..

[30] Abramkin D.S., Putyato M.A., Budennyy S.A., Gutakovskii A.K., Semyagin B.R., Preobrazhenskii V.V., Kolomys O.F., Strelchuk V.V., Shamirzaev T.S. (2012). Atomic structure and energy spectrum of Ga(As,P)/GaP heterostructures. J. Appl. Phys..

[31] Shamirzaev T.S. (2018). Exciton recombination and spin dynamics in indirect-gap quantum wells and quantum dots. Phys. Solid State.

[32] Shamirzaev T.S., Abramkin D.S., Gutakovskii A.K., Putyato M.A. (2012). Novel self-assembled quantum dots in the GaSb/AlAs Heterosystem. JETP Lett..

[33] Abramkin D.S., Rumynin K.M., Bakarov A.K., Kolotovkina D.A., Gutakovskii A.K., Shamirzaev T.S. (2016). Quantum Dots Formed in InSb/AlAs and AlSb/AlAs Heterostructures. JETP Lett..

[34] Shamirzaev T.S., Abramkin D.S., Gutakovskii A.K., Putyato M.A. (2010). High quality relaxed GaAs quantum dots in GaP matrix. Appl. Phys. Lett..

[35] Shamirzaev T.S., Abramkin D.S., Nenashev A.V., Zhuravlev K.S., Trojanek F., Dzurnak B., Maly P. (2010). Carrier dynamics in InAs/AlAs quantum dots: Lack in carrier transfer from wetting layer to quantum dots. Nanotechnology.

[36] Bayer M., Ortner G., Stern O., Kuther A., Gorbunov A.A., Forchel A., Hawrylak P., Fafard S., Hinzer K., Reinecke T.L. (2002). Fine structure of neutral and charged excitons in self-assembled In(Ga)As/(Al)GaAs quantum dots. Phys. Rev. B.

[37] Paillard M., Marie X., Renucci P., Amand T., Jbeli A., Gérard J.M. (2001). Spin relaxation quenching in semiconductor quantum dots. Phys. Rev. Lett..

[38] Bir G.L., Pikus G.E. (1974). Symmetry and Strain-Induced Effects in Semiconductors.

[39] Pikus G.E., Bir G.L. (1971). Exchange interaction in excitons in semiconductors. Sov. Phys. JETP.

[40] Smirnov D.S., Zhukov E.A., Yakovlev D.R., Kirstein E., Bayer M., Greilich A. (2020). Spin polarization recovery and Hanle effect for charge carriers interacting with nuclear spins in semiconductors. Phys. Rev. B.

[41] Merkulov I.A., Efros A.L., Rosen M. (2002). Electron spin relaxation by nuclei in semiconductor quantum dots. Phys. Rev. B.

[42] Debus J., Shamirzaev T.S., Dunker D., Sapega V.F., Ivchenko E.L., Yakovlev D.R., Toropov A.I., Bayer M. (2014). Spin-flip Raman scattering of the Γ-X mixed exciton in indirect band gap (In,Al)As/AlAs quantum dots. Phys. Rev. B.

[43] Ivanov V.Y., Shamirzaev T.S., Yakovlev D.R., Gutakovskii A.K., Owczarczyk S., Bayer M. (2018). Optically detected magnetic resonance of photoexcited electrons in (In,Al)As/AlAs quantum dots with indirect band gap and type-I band alignment. Phys. Rev. B.

[44] Ivanov V.Y., Tolmachev D.O., Shamirzaev T.S., Yakovlev D.R., Slupinski T., Bayer M. (2021). Optically detected magnetic resonance of indirect excitons in an ensemble of (In, Al, Ga)As/(Al, Ga)As quantum dots. Phys. Rev. B.

[45] Nikolaev I.S., Lodahl P., van Driel A.F., Koenderink A.F., Vos W.L. (2007). Strongly nonexponential time-resolved fluorescence of quantum-dot ensembles in three-dimensional photonic crystals. Phys. Rev. B.

[46] Urbaszek B., Marie X., Amand T., Krebs O., Voisin P., Maletinsky P., Högele A., Imamoglu A. (2013). Nuclear spin physics in quantum dots: An optical investigation. Rev. Mod. Phys..

[47] Kuznetsova M.S., Flisinski K., Gerlovin I.Y., Petrov M.Y., Ignatiev I.V., Verbin S.Y., Yakovlev D.R., Reuter D., Wieck A.D., Bayer M. (2014). Nuclear magnetic resonances in (In, Ga)As/GaAs quantum dots studied by resonant optical pumping. Phys. Rev. B.

[48] Dyakonov M., Marie X., Amand T., Jeune P.L., Robart D., Brousseau M., Barrau J. (1997). Coherent spin dynamics of excitons in quantum wells. Phys. Rev. B.

[49] Smirnov D.S., Mantsevich V.N., Glazov M.M. (2021). Theory of optically detected spin noise in nanosystems. Phys. Usp..

[50] Avdeev I.D., Smirnov D.S. (2019). Hyperfine interaction in atomically thin transition metal dichalcogenides. Nanoscale Adv..

[51] Zhukov E.A., Kirstein E., Smirnov D.S., Yakovlev D.R., Glazov M.M., Reuter D., Wieck A.D., Bayer M., Greilich A. (2018). Spin inertia of resident and photoexcited carriers in singly charged quantum dots. Phys. Rev. B.

[52] Shumilin A.V., Smirnov D.S. (2021). Nuclear Spin Dynamics, Noise, Squeezing, and Entanglement in Box Model. Phys. Rev. Lett..

